# Identification and prognostic analysis of biomarkers to predict the progression of pancreatic cancer patients

**DOI:** 10.1186/s10020-022-00467-8

**Published:** 2022-04-15

**Authors:** Wei Li, Tiandong Li, Chenguang Sun, Yimeng Du, Linna Chen, Chunyan Du, Jianxiang Shi, Weijie Wang

**Affiliations:** 1 Department of Hematology, The First Affiliated Hospital of Zhengzhou University, Zhengzhou, 450052 Henan China; 2 Department of Hepatobiliary Surgery, The First Affiliated Hospital of Zhengzhou University, Zhengzhou, 450052 Henan China; 3 Laboratory Animal Center, School of Medical Sciences, Zhengzhou University, Zhengzhou, 450052 Henan China; 4 BGI College and Henan Institute of Medical and Pharmaceutical Sciences in Academy of Medical Science, Zhengzhou University, Zhengzhou, 450052 Henan China; 5 The Academy of Medical Science, College of Medical, Zhengzhou University, Zhengzhou, 450052 Henan China; 6 College of Public Health, Zhengzhou University, Zhengzhou, 450000 Henan China

**Keywords:** Pancreatic cancer, Biomarkers, LAMB3, FN1, KRT19, ANXA1

## Abstract

**Background:**

Pancreatic cancer (PC) is a malignancy with a poor prognosis and high mortality. Surgical resection is the only “curative” treatment. However, only a minority of patients with PC can obtain surgery. Improving the overall survival (OS) rate of patients with PC is still a major challenge. Molecular biomarkers are a significant approach for diagnostic and predictive use in PCs. Several prediction models have been developed for patients newly diagnosed with PC that is operable or patients with advanced and metastatic PC; however, these models require further validation. Therefore, precise biomarkers are urgently required to increase the efficiency of predicting a disease-free survival (DFS), OS, and sensitivity to immunotherapy in PC patients and to improve the prognosis of PC.

**Methods:**

In the present study, we first evaluated the highly and selectively expressed targets in PC, using the GeoMxTM Digital Spatial Profiler (DSP) and then, we analyzed the roles of these targets in PCs using TCGA database.

**Results:**

LAMB3, FN1, KRT17, KRT19, and ANXA1 were defined as the top five upregulated targets in PC compared with paracancer. The TCGA database results confirmed the expression pattern of LAMB3, FN1, KRT17, KRT19, and ANXA1 in PCs. Significantly, LAMB3, FN1, KRT19, and ANXA1 but not KRT17 can be considered as biomarkers for survival analysis, univariate and multivariate Cox proportional hazards model, and risk model analysis. Furthermore, in combination, LAMB3, FN1, KRT19, and ANXA1 predict the DFS and, in combination, LAMB3, KRT19, and ANXA1 predict the OS. Immunotherapy is significant for PCs that are inoperable. The immune checkpoint blockade (ICB) analysis indicated that higher expressions of FN1 or ANXA1 are correlated with lower ICB response. In contrast, there are no significant differences in the ICB response between high and low expression of LAMB3 and KRT19.

**Conclusions:**

In conclusion, LAMB3, FN1, KRT19, and ANXA1 are good predictors of PC prognosis. Furthermore, FN1 and ANXA1 can be predictors of immunotherapy in PCs.

**Supplementary Information:**

The online version contains supplementary material available at 10.1186/s10020-022-00467-8.

## Introduction

Pancreatic cancer (PC) is a highly malignant cancer of the gastrointestinal tract that is characterized by late diagnosis, limited treatment success and a poor prognosis (Chen et al. 2021). The morbidity and mortality of PC continues to rise and constitutes a major challenge for basic and clinical oncologists (Chen et al. 2021). To date, surgical excision remains the only method with curative potential (Strobel et al. 2019). However, at the time of diagnosis, approximately 80–85% of patients have already advanced to either an unresectable or a metastatic state, which accounts for the 5-year survival rate of less than 10% of patients (Mizrahi et al. 2020). Even for the minority of patients who have the opportunity to undergo surgery, the prognosis remains poor, with only 20% surviving for 5 years (Mizrahi et al. 2020). Therefore, there is an urgent need to explore new biomarkers for a clinically meaningful impact on the screening of patients with high-risk PC.

Several studies have demonstrated that the carbohydrate antigen 19-9 (CA 19-9) and the carcinoembryonic antigen (CEA) can be used to predict the outcomes in a variety of cancers, including PC (Stojkovic Lalosevic et al. 2017; Zhou et al. 2017; Distler et al. 2013; Liu et al. 2018a). However, these biomarkers are not sufficiently specific or sensitive for use in PC (Zhu et al. 2017; Swords et al. 2016). In addition, high-throughput sequencing has identified a large number and different varieties of biomarkers; however, few hold future promise as a preferred marker for PC (Ballehaninna and Chamberlain 2013). This is because most of these discovered biomarkers have significant limitations, such as a lack of methodological standardization and quality control; limited or no correlation with tumor stage and tumor invasives; and minimal utility to assess prognosis, predict tumor recurrence, or evaluate tumor immunotherapy response (Ballehaninna and Chamberlain 2013; Nixon et al. 2013; Sahni et al. 2020; Pereira et al. 2020).

PC is a special kind of tumor; the number of tumors that are operable are small, and the paraneoplastic and cancerous tissues are not easily obtained or distinguished from cancerous tissue (Nixon et al. 2013). Pathology testing often requires the use of as few or limited tissue samples as possible, while testing for multiple biomarkers. High-plex profiling of gene expression under bulk or single-cell analysis provides rich contextual information but consumes large fractions of tissue and lacks spatial context of the key morphological features (Sorg et al. 2020). As such, direct interrogation tools are required to enable characterization of localized transcriptomic changes in discrete tissue while preserving additional tissue for testing. The GeoMxTM Digital Spatial Profiling (DSP) platform robustly quantifies high-plex RNA expression data from single, 5 μm sections, capturing genome-wide expression patterns in spatially resolved locations throughout the tissue, and it has already been used to study many types of cancers (Van and Blank 2019; Chandramohan et al. 2021; Gong et al. 2020; Kalita-de Croft et al. 2021). In this article, we present the DSP method to study PC. The GeoMx Cancer Transcriptome Atlas (CTA) targets are designed for comprehensive profiling of tumors, tumor microenvironment, and tumor immune status with 1833 RNA targets, including negative controls. We conducted 1,833 RNA targets on both cancer and paracancer tissues, and then we identified differentially expressed genes between cancer and paracancer tissue samples. We then confirmed the expression pattern of the identified differentially expressed markers with the Cancer Genome Atlas (TCGA) data set. Survival analysis, the univariate and multivariate Cox proportional hazards model, risk model analysis and ICB responses analysis were used to confirm the predictive roles of these markers in PCs. Our approach for combining these two powerful technologies has the potential to provide meaningful biological insight in PCs.

## Materials and methods

### Fresh frozen section preparation and hematoxylin and eosin (H&E) staining

Cancer and paracancer tissue samples were obtained from the same patient during surgery and immediately embedded into an optimal cutting temperature (OCT) compound. Thereafter, H&E staining was performed, as previously described (Cao et al. 2022). Briefly, the frozen sections are allowed to warm at room temperature for 5–10 min, and then the rewarmed slices are soaked in water for approximately 30–60 s. The tissues are immersed in distilled water for 1–2 min, while ensuring that the distilled water covers the tissues entirely and that the water is evenly distributed. The tissues are then stained with hematoxylin staining solution for 5 min and washed with water for 3–5 s. This is followed by staining with eosin for 2 min, washing with distilled water for 1–2 min, and blotting with filter paper or natural drying. Finally, CaseViewer software was used to analyze the results of the cancer and paracancer tissue samples.

### Immunofluorescence (IF)

The frozen sections were removed and allowed to dry at room temperature for 15 min. They were then soaked in PBS solution for 10 min to remove the OCT compound. The tissue to be stained was circled with a histochemical grease pencil to allow effective incubation of the antibodies. The sections were permeabilized with 0.5% TritonX-100 at room temperature for 20 min and closed with PBS solution containing 10% normal goat serum for 1 h at room temperature to improve the accuracy of the target protein and to reduce the background. The antibodies CD45-Alexa 594 and PanCK-488 were added, and the sections were incubated at 4 °C overnight. Then, PBS washed three times for 10 min each. Syto 13 was incubated for in the dark for 5 min to stain the nuclei and PBS washed three times for 10 min each. Finally, the fluorescence microscope was used to photograph the results.

### GeoMx DSP for CTA profiling

GeoMx Cancer Transcriptome Atlas Panel was used in this study and fresh surgically excised cancerous and paracancerous tissue samples were immediately embedded in OCT compound to prepare frozen sections. Then, the fresh-frozen tissues were processed, following the GeoMx DSP slide prep user manual as previously described (Desai et al. 2020). Briefly, (1) RNA target exposure: proteinase K was added prior to the incubation with RNA probe mix (CTA panel). (2) Staining: after overnight incubation, the slides were washed with buffer and stained with CD45-Alexa 594, PanCK-488, and SYTO 13 for 1 h and loaded into the GeoMx DSP machine to scan the fluorescent images. (3) Regions of interest (ROIs) selection: ROIs were placed by aligning to those ROIs identified during protein profiling. (4) Sequencing library construction: oligos were cleaved and collected into 96-well plates. Oligos from each area of interest (AOI) were uniquely indexed using Illumina’s i5 × i7 dual-indexing system. Four μL of a GeoMx DSP sample was used in the PCR reaction. The PCR reactions were purified with two rounds of AMPure XP beads (Beckman Coulter) at a 1:2 bead-to-sample ratio. (5) Sequencing: Libraries were paired-end sequenced (2 × 75) on a NextSeq550 up to 400 M total aligned reads. (6) Analytical process: Fastq files were processed by the NanoString DND pipeline to generate count files for each target probe and saved as DCC files.

### GeoMx RNA data quality control (QC)

GeoMx DSP data analysis software was used for primary analysis and then referring to annotation information included in Additional file 3: Table S1 for data interpretation. Three methods of QC were used in the present study. (1) Technical signal QC is a sequencing quality evaluation for each AOI/ROI with three metrics: raw reads, aligned reads percentage, and sequencing saturation. Raw reads include all the reads sequences of AOI/ROI at the time of sequencing. Aligned reads percentage is the percentage of reads sequences in the AOI/ROI that are compared to the template sequences. Sequencing saturation are the sequencing reads of an AOI/ROI that can be sequenced once or more times; sequencing saturation is the percentage of reads that are detected at least twice in raw reads, and it is recommended that it should exceed 50%.

(2) Technical background QC is a running comparison of GeoMx DSP settings, divided into two metrics, no template control count (NTC count) and negative probe count. NTC count is a negative control experiment set up during each CTA experiment, and it does not contain a template for detecting template contamination during library construction. The NTC value for this CTA experiment is 3000. Negative probe count is the number of negative probes in each AOI/ROI, which is used to measure the technical noise level of the CTA experiment. Low technical noise requires consideration of whether the AOI/ROI area is too small, whether the library build is successful, and whether the sequencing depth is too low. Based on the empirical value of the negative probe measurement, a technical noise of five or more is required for each CTA experiment. GeoMx DSP will limit the nuclei counts and surface area for each AOI/ROI, and too low nuclei counts or surface area is not recommended.

(3) Probe QC includes two aspects, namely, probe outlier detection and target LOQ detection. Target is the gene to be detected, and GeoMx DSP will design multiple probes to detect each target. LOQ is the limit of quantitation, and, if a target's count value is below LOQ, it does not mean it is not expressed, while, if it is greater than LOQ, its expression can be confirmed. LOQ represents the limit value of a target confirmation expression, which is calculated as follows: LOQ = GeoMean (NegProbe) × GeoSD (NegProbe)^threshold^. In CTA, a threshold value of 2.5 represents a more stringent standard, while a threshold value of 2.0 represents a slightly more lenient standard. In this CTA experiment, the LOQ value was 102 and the threshold value 2.0. GeoMx DSP was designed with multiple probes for target genes, and, if a probe performed too abnormally, it was rejected. There are two rejection indicators, low outlier detection and Grubb’s outlier test. Low outlier detection is defined as low outlier if the count value of a probe is less than 10% of the average count value of other probes. Grubb’s outlier test was performed for all probes in each target. If a probe was marked as an outlier in more than 20% of the AOI/ROI, this probe was removed.

### Differential expression analysis of GeoMx RNA data

For each tissue and each gene, differential expression versus cancer presence/absence was evaluated with an unpaired, heteroscedastic t-test of the gene’s log2-transformed normalized data. For analysis of expression within the tissues, genes were defined as consistently upregulated if they had (1) a log2 fold change > 0.2 and a Benjamini–Hochberg FDR < 0.1 in at least 2 ROIs, and (2) never had a log2 fold change < 0 and a Benjamini–Hochberg FDR < 0.1 in any ROI. Genes were defined as consistently downregulated by an equivalent rule. For analysis across patient samples, expression was modeled using linear mixed effect models that allow for random slope and intercept terms per patient sample. *P*-values were estimated with Satterthwaite’s method for approximation and adjusted with the Benjamini–Hochberg FDR. Only genes that rose twofold above-background in at least one ROI were considered.

### Differentially expressed genes analysis and KEGG pathways analysis of PCs from the TCGA database

Tumoral RNA-seq data for the 178 PC patients on the TCGA database were downloaded from the Genomic Data Commons (GDC) data portal (TCGA), and four of the tumors also had RNA-seq data of paired normal tissue samples. All data of normal tissue samples were obtained from 328 pancreases in GTEx V8 release version (https://gtexportal.org/home/datasets). The limma package of R software was used to analyze the differentially expressed genes as our previously described (Cao et al. 2022; Li et al. 2019; Wang et al. 2021). Briefly, “Adjusted *P* < 0.05 and Log2 (FC) > 1 or Log2 (FC) < − 1” were defined as the thresholds for the screening of differentially expressed genes. To better understand the carcinogenesis of mRNA, the clusterProfiler package in R software was employed to analyze the enrich the Kyoto Encyclopedia of Genes and Genomes (KEGG) pathway.

### Kaplan–Meier curves, univariate and multivariate Cox proportional hazards model and risk model analysis

RNA-sequencing expression profiles and corresponding clinical information for PC patients were downloaded from the TCGA dataset (https://portal.gdc.com). The Kaplan–Meier curves, univariate and multivariate Cox proportional hazards model, and risk model analysis (Lasso cox regression) based on the expression of LAMB3, FN1, KRT19, and ANXA1 were constructed, as previously described (Lin et al. 2020). Disease-free survival time (DFS) and Overall-survival time (OS) were compared between the high and low LAMB3, FN1, KRT19, and ANXA1 risk groups via a Kaplan–Meier analysis, using the survival and survminer packages in R. The cutoff value for the high and low risk was defined as the median value of LAMB3, FN1, KRT19, or ANXA1. A univariate Cox analysis was performed to identify potential prognostic factors, and a multivariate Cox analysis was used to determine risk score as an independent risk factor for OS in PC. A ROC curve was generated to validate the accuracy of the risk model in predicting patients’ DFS and OS via the survival ROC R package.

### Immune checkpoint blockade (ICB) response analysis

Raw counts of RNA-sequencing data (level 3) and corresponding clinical information from 178 PC patients were obtained from The TCGA dataset (https://portal.gdc.cancer.gov/), in which the methods of acquisition and application complied with the guidelines and policies. Potential ICB response was predicted with a TIDE algorithm, as previously described (Jiang et al. 2018). Briefly, TIDE uses a set of gene expression markers to assess 2 different mechanisms of tumor immune escape, including dysfunction of tumor-infiltrating cytotoxic T lymphocytes (CTL) and exclusion of CTL by immunosuppressive factors. High TIDE scores are associated with poor efficacy of immune checkpoint blockade therapy (ICB) and short survival after receiving ICB therapy.

### Statistics analysis

R version 4.0.3. software was used for all statistical analyses. The statistical details of all experiments are reported in the materials and methods, including statistical analysis performed and statistical significance.

## Results

### Selection ROIs between pancreatic cancer and paracancer

**Fig. 1:**
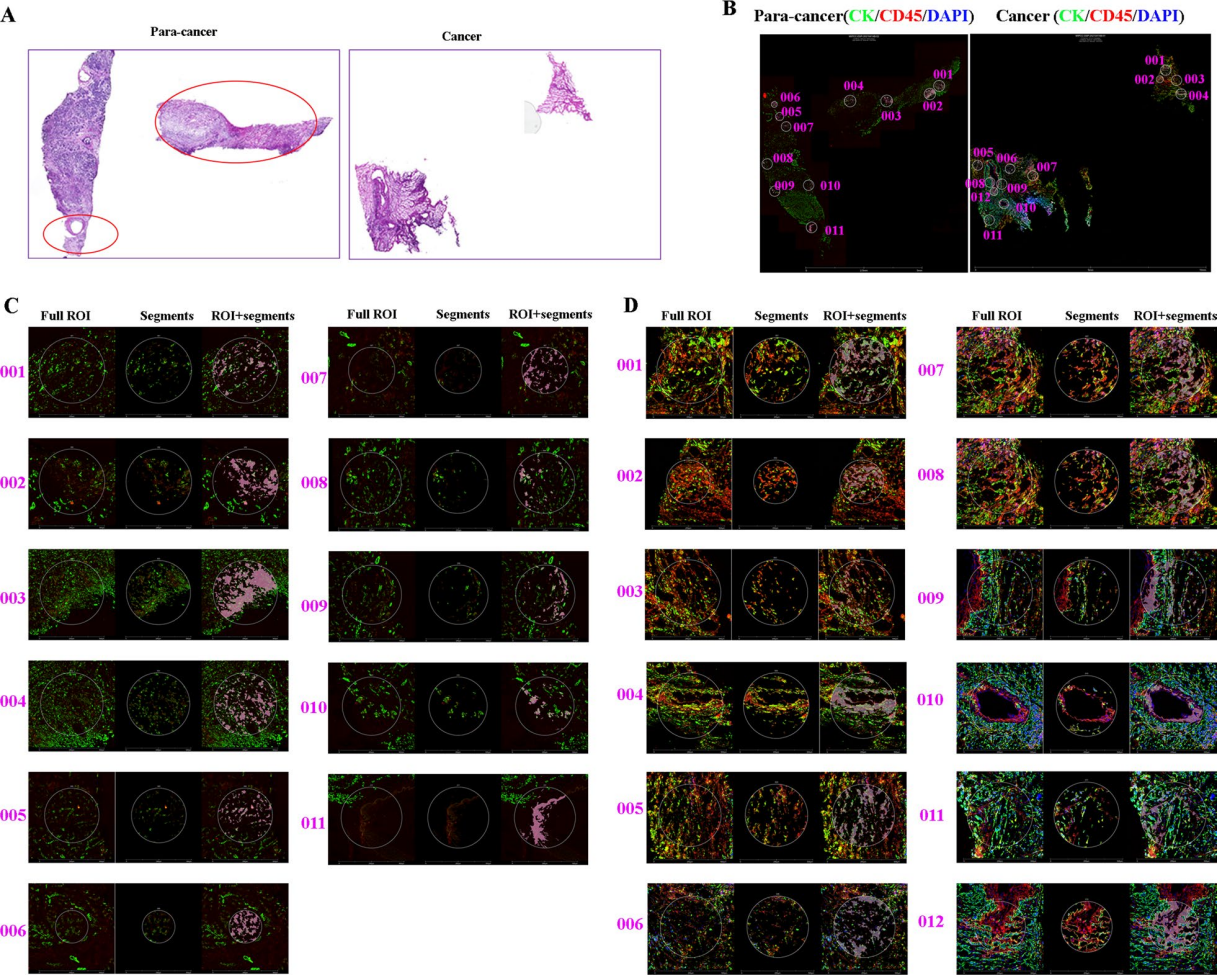
**A** Paracancer and cancer samples from a PC patient as confirmed by H&E staining. **B** Paracancer and cancer samples from a PC as assessed by immunofluorescence (IF) staining to select appropriate ROIs. Green for CK staining, red for CD45 staining, and blue for Syto 13 staining. **C** The selected ROIs shown in the paracancer sample. **D** The selected ROIs shown in the cancer sample

To generate unbiased transcriptomic maps of the PC tissue sections, we mounted cryosections of freshly frozen cancer tissue and paracancer tissue that originated from the same patient onto the spatially barcoded microarray slides. The age of this patient is 61-years old with ECOG score of 0. The patient was received surgery at March, 22, 2021. And the patient's pathological type is a medium- to low-differentiated adenocarcinoma of the tail of the pancreatic body. The TNM stage of this patient is stage II. Then, we processed the freshly frozen cancer tissue and paracancer tissue by H&E staining (Fig. 1A). Although the two tissue sections are from cancer tissue and paracancer tissue, the paracancer tissue circled in red resembles cancerous tissue morphologically, indicating that paracancer tissues contain normal pancreatic tissue and cancer tissue. To obtain ROIs from the two tissue samples, we stained them with CK, CD45, and Syto 13 (Fig. 1B). In the paracancer tissue, we selected 11 ROIs (Fig. 1C), which included five cancer tissues (001, 002, 003, 004, and 011), and six normal tissues (005, 006, 007, 008, 009, and 010). In the cancer tissue sample, we selected 12 ROIs (Fig. 1D, 001–012). The ROIs were then processed for CTA analysis, including complementary DNA synthesis, amplification by in vitro transcription, library construction, and sequencing, as previously described (Chandramohan et al. 2021). The GeoMx CTA Panel was used to analyze the expression pattern of the two sections.

### Comparison of the differentially expressed genes between cancer and paracancer tissues by DSP analysis

**Fig. 2:**
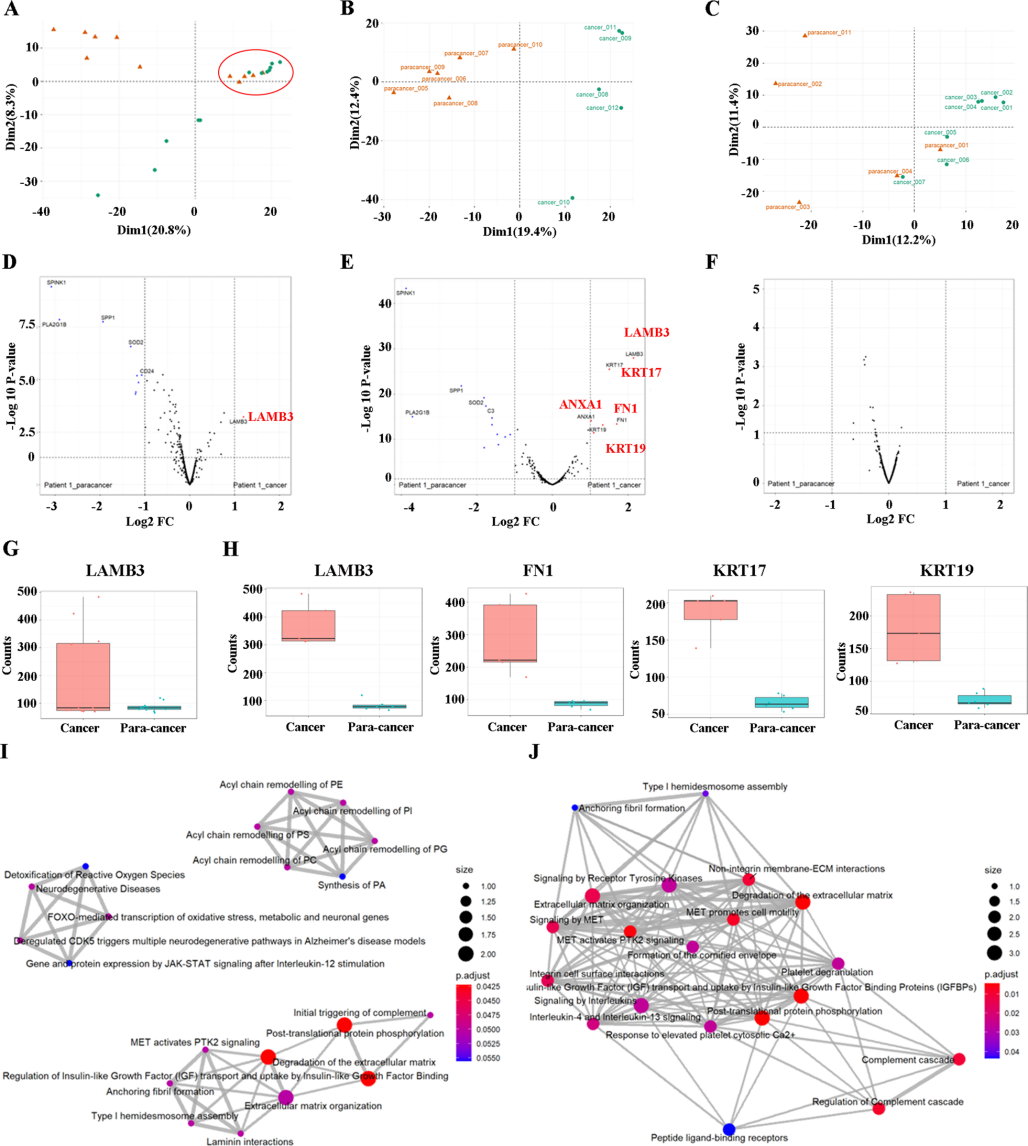
**A** Principal component analysis (PCA) of DSP data demonstrate paracancer ROIs 001–011 and cancer ROIs 001–012. **B** Principal component analysis (PCA) of DSP data demonstrates paracancer ROIs 005–010 and cancer ROIs 008–012. **C** Principal component analysis (PCA) of DSP data demonstrates paracancer ROIs 001–004 and 011 and cancer ROIs 001–007. **D** Volcano map display of the differentially expressed genes between paracancer ROIs 001–011 and cancer ROIs 001–012. **E** Volcano map display of the differentially expressed genes between paracancer ROIs 005–010 and cancer ROIs 008–012. **F** Volcano map display of the differentially expressed genes between paracancer ROIs 001–004 and 011 and cancer ROIs 001–07. **G** The mean expression level of upregulated target of LAMB3 in paracancer ROIs 005–010 and cancer ROIs 008–012. **H** The mean expression level of upregulated target of LAMB3, FN1, KRT17, and KRT19 in paracancer ROIs 005–010 and cancer ROIs 008–012. **I** The pathway network in cancer ROIs 001–012. **J** The pathway network in cancer ROIs 008–012

We then analyzed the data with the GeoMx Digital Spatial Profiler (DSP). To begin, we performed quality control (QC) on the data. Segment QC was performed to quality control the area of interest (AOI)/ROI, which includes technical signal QC, technical background QC, and GeoMx DSP parameters. Additional file 1: Fig. S1A–C shows raw reads, aligned reads percentage, sequencing saturation, negative probe count, nuclei counts, and surface area. We next performed the probe QC, which includes two aspects, probe outlier detection and the target LOQ detection. Additional file 1: Fig. S1D and E demonstrate the low outlier detection, Grubbs outlier test, and the LOQ. All the metrics demonstrate satisfactory sequencing quality. Thereafter, we used an unbiased tSNE clustering of all ROIs and identified three distinct clusters (Fig. 2A). The cluster circled in red contains the ROIs 001, 002, 003, 004, and 011 from the paracancer tissue, which resembles cancerous tissue morphologically. The other ROIs from the cancer tissue, 001–007, are also contained in this cluster. This suggests that these ROIs from the two tissue samples are similar, which is consistent with H&E staining. We then performed PCA clustering separately for those with and without differences in ROIs again to compare the differentially expressed genes (Fig. 2B and C). In the present study, we selected genes with Log2 FC > 1 as significantly differentially expressed. Figure 2D demonstrates the significantly upregulated and downregulated genes between all the ROIs from the paracancer tissue and the cancer tissue. Figure 2E illustrate the significantly upregulated and downregulated genes between ROIs with differences. However, there are no significant differentially expressed genes between ROIs without differences (Fig. 2F). Consistent with PCA analysis, similar ROIs showed no differentially expressed genes, which further confirms that ROIs 001,002,003,004, and 011 from the paracancer tissue are cancer tissue. Then, we analyzed the upregulated top five differentially expressed genes between the paracancer tissue and the cancer tissue, which included LAMB3, FN1, KRT17, KRT19, and ANXA1 (Fig. 2G and H). All the expression of the 1833 targets from the paracancer tissue and the cancer tissue are shown in Additional file 4: Table S2. The KEGG pathway enrichment analysis of the cancer tissue included laminin interactions, degradation of the extracellular matrix, extracellular matrix organization, and more, all of which were highly associated with the upregulated genes (Fig. 2I and J). Taken together and using the GeoMx CTA Panel, we identified five highly and selectively expressed genes in the PC tissues.

### Survival analysis demonstrated distinct prognostic effects of LAMB3, FN1, KRT17, KRT19, and ANXA1 in pancreatic cancer

**Fig. 3:**
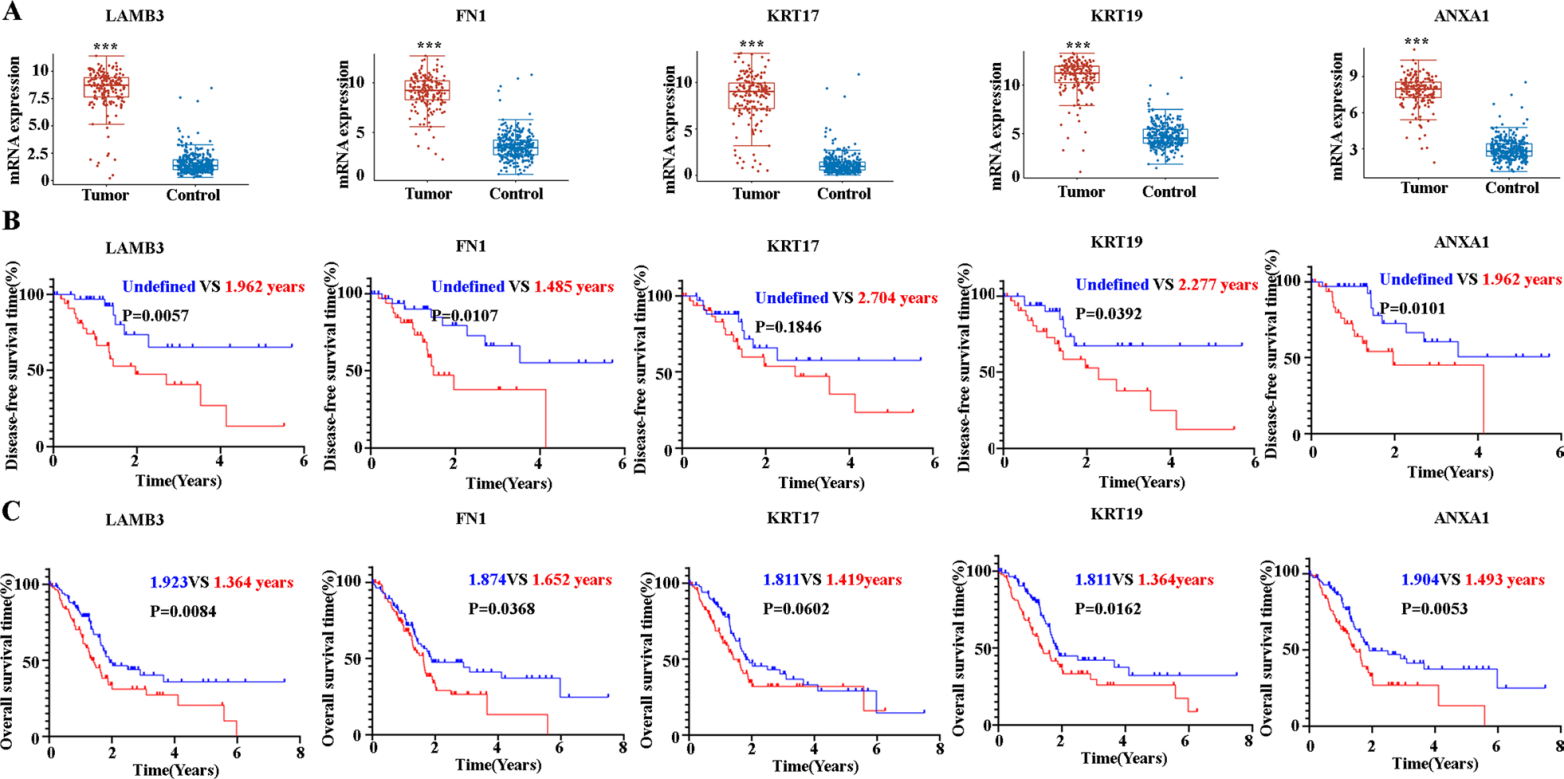
**A** The mRNA expression levels of LAMB3, FN1, KRT17, KRT19, and ANXA1 as assessed by RNA-seq between PCs and controls (including paracancer or normal pancreas). **B** The Kaplan–Meier DFS curves for PC patients assigned to high and low expression groups of LAMB3, FN1, KRT17, KRT19, and ANXA1 based on the expression level, respectively. The median was selected as the cutoff value for high or low groups. The blue lines are the low expression groups and the red lines are the high expression groups. **C** The Kaplan–Meier OS curves for PC patients assigned to high and low expression groups of LAMB3, FN1, KRT17, KRT19, and ANXA1 based on the expression level, respectively. The median was selected as the cutoff value for high or low groups. The blue lines are the low expression groups and the red lines are the high expression groups

The expression pattern of LAMB3, FN1, KRT17, KRT19, and ANXA1 was then confirmed in 178 PC patients (Stage I: 24 patients, Stage II: 146, Stage III: 3, Stage IV: 5) from the TCGA database. Compared with the adjacent tissues (N = 4) and normal tissues (N = 328), LAMB3, FN1, KAR17, KAR19, and ANXA1 were significantly upregulated (Fig. 3A). The Kaplan Maier survival analysis with log-rank tests was used to compare the difference in DFS and OS between high and low expression of LAMB3, FN1, KAR17, KAR19 and ANXA1. Sixty-nine of the 178 patients had undergone surgery, and we analyzed the DFS based on those 69 patients. The analysis of OS was based on all 178 patients. It is noteworthy that the analysis indicated that the LAMB3^hi^, FN1^hi^, KRT19^hi^, and ANXA1^hi^ groups demonstrated shorter DFS (undefined VS. 1.962 years, *P* = 0.0057; undefined VS. 1.485 years, *P* = 0.0107; undefined VS. 2.277 years, *P* = 0.0392; undefined VS. 1.962 years, *P* = 0.0101) and OS (1.923 years vs 1.364 years, *P* = 0.0084; 1.874 years VS 1.652 years, *P* = 0.0368; 1.811 years vs 1.364 years, *P* = 0.0162; 1.904 years VS 1.493 years, *P* = 0.0054). In contrast, there were no significant differences for both DFS and OS based on the high and low expression of KRT17 (Fig. 3B and C).

### Univariate analysis and multivariate Cox proportional hazards model

**Fig. 4:**
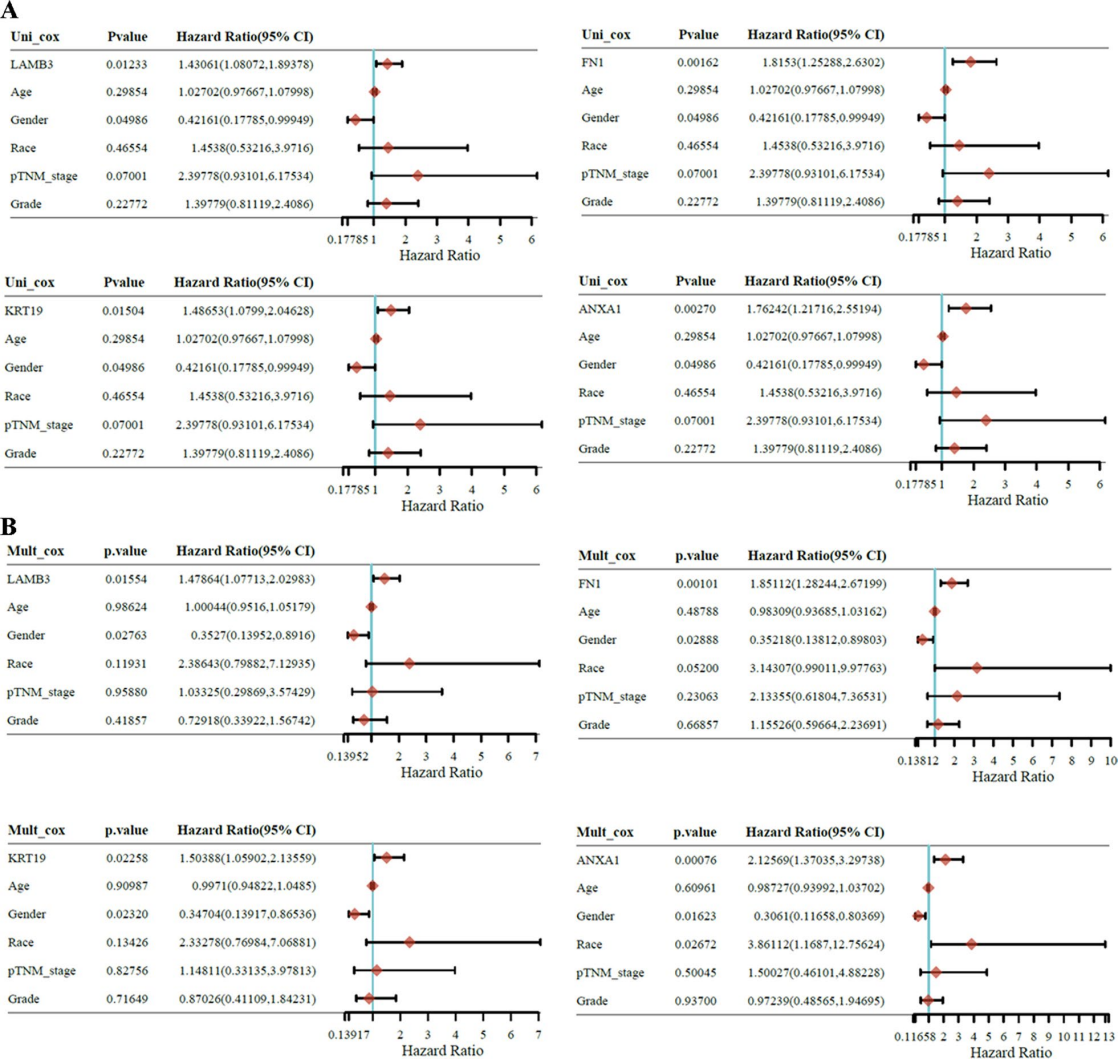
**A** The univariate analysis revealing the hazard ratio, *P*‐values, and some parameters of LAMB3, FN1, KRT19, and ANXA1 in terms of DFS in PC patients. **B** The multivariate Cox proportional hazards model revealing the hazard ratio, *P*‐values, and some parameters of LAMB3, FN1, KRT19, and ANXA1 in terms of DFS in PC patients

**Fig. 5:**
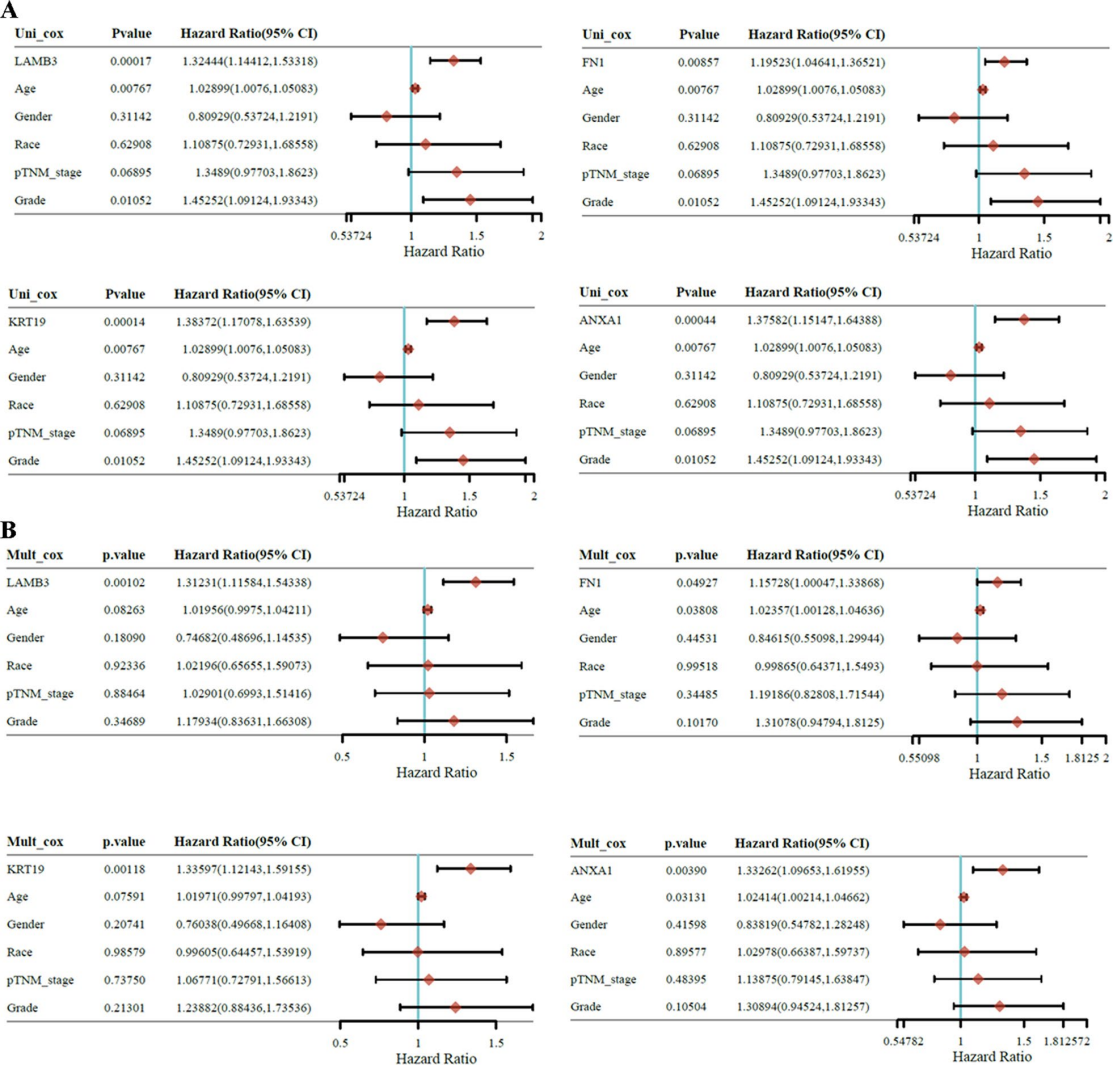
**A** The univariate analysis evaluating the hazard ratio, *P*‐values, and some parameters of LAMB3, FN1, KRT19, and ANXA1 in terms of OS in PC patients. **B** The multivariate Cox proportional hazards model evaluating the hazard ratio, *P*‐values, and some parameters of LAMB3, FN1, KRT19, and ANXA1 in terms of OS in PC patients

Subsequently, we conducted univariate and multivariate Cox analyses to evaluate the independent prognostic value of LAMB3, FN1, KRT19, and ANXA1 in terms of both DFS and OS of patients with PC. The univariate analysis indicated that the group with high LAMB3, FN1, KRT19, and ANXA1 were significantly correlated with shorter DFS (Fig. 4A) and OS (Fig. 5A). Multivariate analyses revealed that the group with high LAMB3, FN1, KRT19, and ANXA1 were still independently associated with significantly poorer DFS (Fig. 4B) and OS (Fig. 5B) of patients with PC, which could serve as independent prognostic factors for PC.

### Construction and validation of a risk model according to LAMB3, FN1, KRT19, and ANXA1 in PC patients

**Fig. 6:**
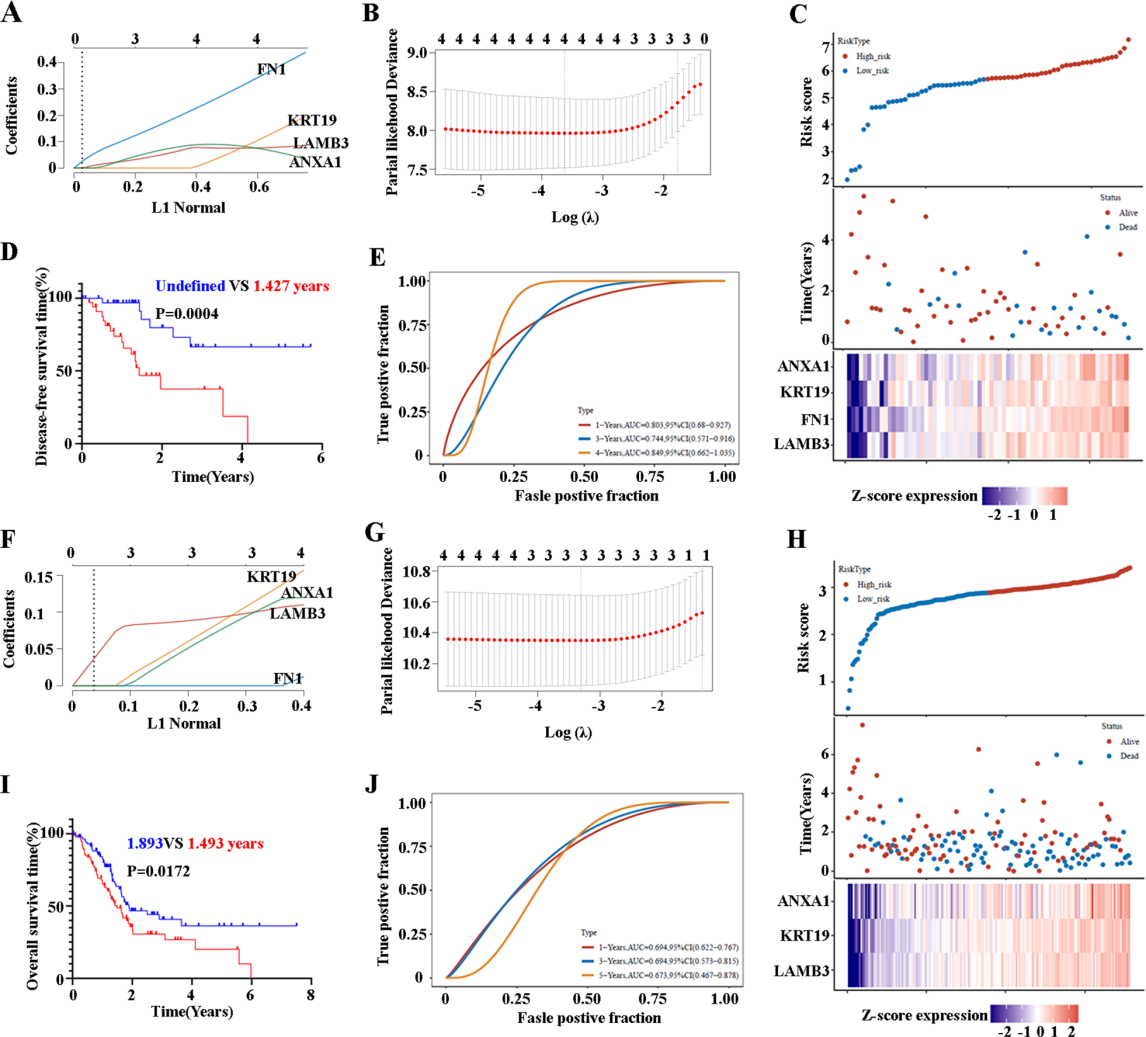
**A** LASSO coefficients profiles of LAMB3, FN1, KRT19, and ANXA1 are shown by lambda parameter in relation to DFS. **B** LASSO Cox regression with ten-fold cross-validation obtained using minimum lambda value related to DFS. **C** Prognostic analysis of LAMB3, FN1, KRT19, and ANXA1 signatures in the TCGA set related to DFS. Top: The dotted line represents the median risk score and divides the patients into low-risk and high-risk groups. Middle: Survival status of the patients are shown in red (alive) and blue (dead) dots. Down: Heatmap of the expression profiles of the four prognostic genes (LAMB3, FN1, KRT19, and ANXA1) in the low- and high-risk groups relating to DFS. **D** The Kaplan–Meier DFS curves for PC patients assigned to low-risk and high-risk groups. The blue line represents the low-risk group and the red line the high-risk group. **E** ROC curves showing the predictive efficiency of the LAMB3, FN1, KRT19, and ANXA1 signatures relating to DFS on the 1-year, 3-year, and 4-year survival rate. **(F)** LASSO coefficients profiles of LAMB3, FN1, KRT19, and ANXA1 are shown by lambda parameter relating to OS. **G** LASSO Cox regression with ten-fold cross-validation obtained using minimum lambda value related to OS. **H** Prognostic analysis of LAMB3, FN1, KRT19, and ANXA1 signatures in the TCGA set relating to OS. The dotted line represents the median risk score and divides the patients into a low-risk and a high-risk group. Survival status of the patients are shown in red (alive) and blue (dead) dots. Heatmap of the expression profiles of the prognostic genes (LAMB3, FN1, KRT19, and ANXA1) showing in the low- and high-risk groups related to OS. **(I)** The Kaplan–Meier OS curves for PC patients assigned to the low-risk and high-risk groups. The blue line represents the low-risk group and the red line the high-risk group. **J** ROC curves showing the predictive efficiency of the LAMB3, FN1, KRT19, and ANXA1 signatures relating to OS on the 1-year, 3-year, and 4-year survival rate

Next, we constructed and validated a risk model (LASSO Cox regression), according to the expression of LAMB3, FN1, KRT19, and ANXA1 in PC patients, which can effectively discern most of the available forecast markers and produce prognostic indicators for predicting clinical results. We first analyzed the prognostic effects of LAMB3, FN1, KRT19, and ANXA1 for predicting the DFS of PC patients. The coefficients of the selected features of LAMB3, FN1, KRT19, and ANXA1 are shown through the lambda parameter (Fig. 6A). Partial likelihood deviance versus log (λ) was drawn using the LASSO Cox regression model (Fig. 6B). Hence, LAMB3, FN1, KRT19, and ANXA1 were selected for the subsequent multivariate analysis. And the risk score of every patient was calculated as previously described (Lin et al. 2020). PC patients were categorized into low-risk group (n = 89) and high-risk group (n = 89), based on the median cut-off point obtained by “survminer” R package analysis (Fig. 6C top). The survival status and survival time of patients in the two different risk groups are shown in the Fig. 6C middle, and the relative expression standards of LAMB3, FN1, KRT19, and ANXA1 are presented in Fig. 6C down. The survival analysis demonstrated that the DFS of the high-risk group was overall shorter than that of the low-risk group (*P* = 0.0004; Fig. 6D). The AUC was 0.803 at 1 year, 0.744 at 3 years, and 0.849 at 5 years, indicating the high predictive value of combined LAMB3, FN1, KRT19, and ANXA1 (Fig. 6E). Using the same analysis method, we next examined the prognostic effects of LAMB3, FN1, KRT19, and ANXA1 for predicting the OS of PC patients, and only LAMB3, KRT19, and ANXA1 were selected for the multivariate analysis for the OS (Fig. 6F and G). Figure 6H shows the two risk groups, based on the expression of combined LAMB3, KRT19, and ANXA1, and Fig. 6I shows that the low-risk group had a longer OS compared with the high-risk group. The AUC was 0.694 at 1 year, 0.694 at 3 years, and 0.673 at 5 years, indicating the predictive value of combined LAMB3, KRT19, and ANXA1 for the OS of PC patients (Fig. 6J).

### Immune checkpoint blockade (ICB) response analysis of PC patients, based on the high and low expression of LAMB3, FN1, KRT19, and ANXA1

**Fig. 7:**
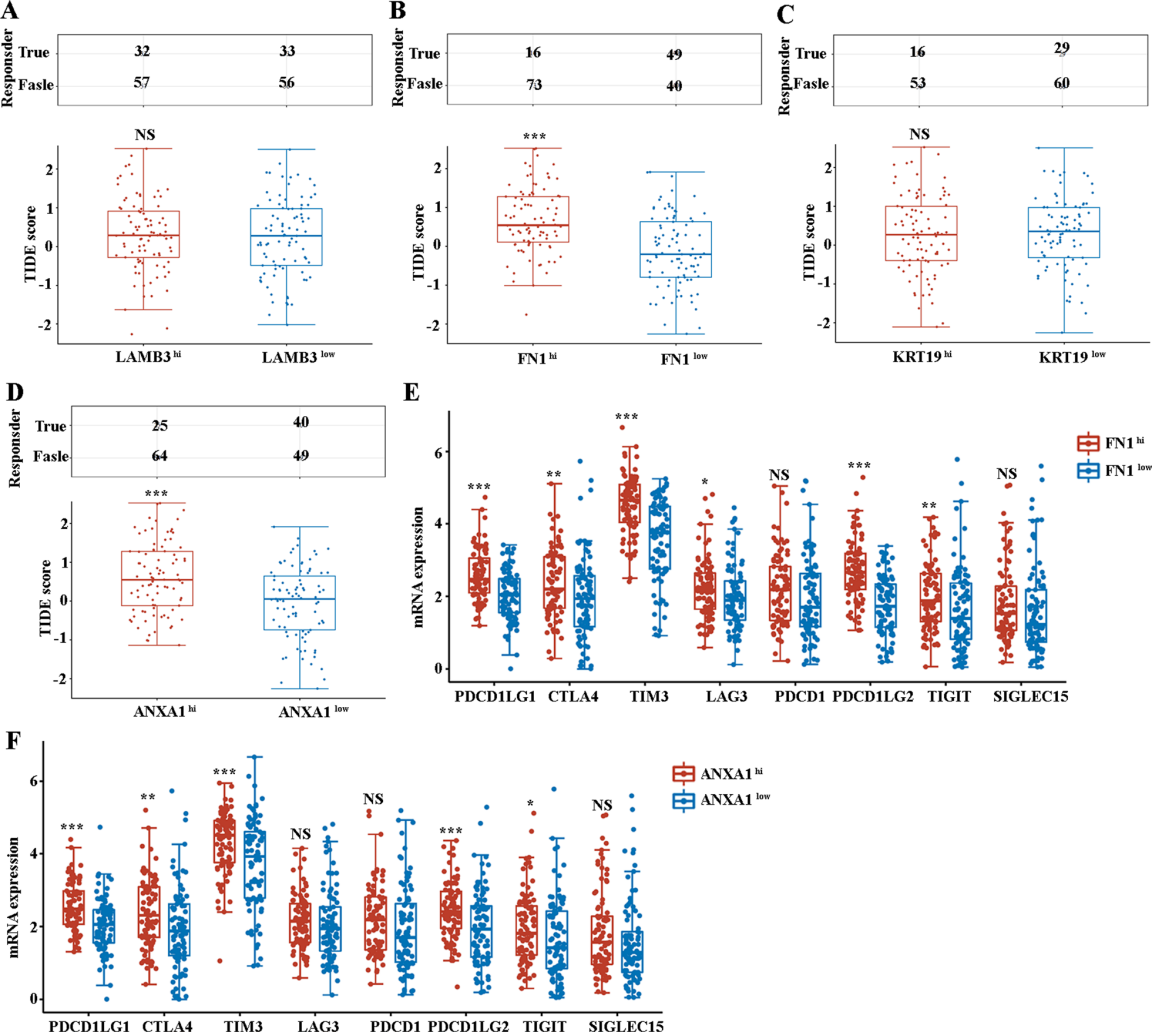
**A** The TIDE score for PC patients showing in the LAMB3^hi^ and LAMB3^low^ groups. **B** The TIDE score for PC patients showing in the FN1^hi^ and FN1^low^ groups. **C** The TIDE score for PC patients showing in the KRT19^hi^ and KRT19^low^ groups. **D** The TIDE score for PC patients showing in the ANXA1^hi^ and ANXA1^low^ groups. **E** The mRNA expression of immune checkpoints for PC patients showing in the FN1^hi^ and FN1^low^ groups. **F** The mRNA expression of immune checkpoints for PC patients showing in the ANXA1^hi^ and ANXA1^low^ groups

Cancer treatment with ICB can provide long-lasting clinical benefits, but only a fraction of patients responds to this treatment. Tumor immune dysfunction and exclusion (TIDE) were used to predict the potential ICB response in previous studies (Jiang et al. 2018; Wang et al. 2020). We evaluated the TIDE scores, based on the high and low expression of LAMB3, FN1, KRT19, and ANXA1, in 89 PC patients undergoing ICB treatment. Remarkably, high expressions of FN1 or ANXA1 aligned with higher TIDE scores, compared with low expression of FN1 (Fig. 7B) or ANXA1 (Fig. 7D). Significantly, higher TIDE scores were correlated to lower ICB response. In contrast, there were no significant differences in TIDE scores based on the expression of LAMB3 (Fig. 7A) or KRT19 (Fig. 7C). Next, we compared the expression of immune checkpoints based on the expression of FN1 or ANXA1. Figure 7E indicated that FN1hi PC patients have higher PDCD1LG1, CTLA4, TIM3, LAG3, PDCD1LG2, and TIGIT, compared with low expression of FN1. Figure 7F demonstrated similar expression pattern of immune checkpoints for ANXA1hi PC patients. Taken together, FN1 or ANXA1 expression can predict the response of PC patients to ICB.

### The differentially expressed genes and KEGG pathways analysis based on the high and low expression of LAMB3, FN1, KRT19, or ANXA1

Given that LAMB3, FN1, KRT19, and ANXA1 can be considered as prognostic biomarkers for PCs, we finally analyzed the transcriptional expression differences between high and low expression of LAMB3, FN1, KRT19, or ANXA1 in patients with PC. Additional file 2: Fig. S2A demonstrates the 433 upregulated genes and 89 downregulated genes in the LAMB3^hi^ group compared with the LAMB3^low^ group in volcano plots (Additional file 3: Table S1). The enriched KEGG signaling pathways were selected to demonstrate the primary biological actions of the major potential mRNA. Additional file 2: Fig. S2B shows the enrichment of KEGG pathways in the LAMB3^hi^ group, including tight junction, PI3K-Akt signaling pathway, focal adhesion, ECM-receptor interaction, and cell adhesion molecules. Additional file 2: Fig. S2C shows the enrichment of KEGG pathways in LAMB3^low^ group, including pancreatic secretion, protein digestion and absorption, fat digestion and absorption, and drug metabolism-cytochrome P450. Using the same analysis method, we found that there are 387 upregulated genes and seven downregulated genes in the FN1^hi^ group compared with the FN1^low^ group (Additional file 2: Fig. S2D, Additional file 3: Table S3). The enrichment of KEGG pathways in the FN1^hi^ group included the PI3K-Akt signaling pathway, focal adhesion, and ECM-receptor interaction (Additional file 2: Fig. S2E). We then analyzed the differentially expressed genes and pathways between the KRT19^hi^ group and KRT19^low^ group. Four hundred and forty-three upregulated genes and 87 downregulated genes were identified in the KRT19^hi^ group compared with KRT19^low^ group (Additional file 2: Fig. S2F, Additional file 6: Table S4). In addition, the enrichment of KEGG pathways in the KRT19^hi^ group and KRT19^low^ group are similar to the LAMB3^hi^ group and LAMB3^low^ groups (Additional file 2: Fig. S2G and H). Finally, there were 223 upregulated genes and 45 downregulated genes in the ANXA1 ^hi^ group compared with the ANXA1^low^ group (Additional file 2: Fig. S2I, Additional file 7: Table S5). The enrichment of KEGG pathways in the ANXA1^hi^ and ANXA1^low^ groups is presented in Additional file 2: Fig. S2J and K.

## Discussion

PC is a deadly disease with a 5-year survival rate of an approximately 10% in the USA, and it is becoming an increasingly common cause of cancer deaths (Mizrahi et al. 2020). Surgical resection represents the only option for a possible cure, and advancements in adjuvant chemotherapy have improved long-term outcomes in these patients (Mizrahi et al. 2020). In addition, targeted therapy and immunotherapy have increased the OS of advanced and metastatic patients (Strobel et al. 2019). However, PC is still an uncurable malignant disease. Many strategies aimed at finding novel targeted therapy and immunotherapy, such as deconstructing the surrounding desmoplastic stroma and targeting the immunosuppressive pathways have largely failed (Ho et al. 2020). Therefore, considering the functional complexity of PC, finding novel biomarkers to predict the prognosis of PC patients at different stages and to provide personalized treatment measures will be a more rational treatment approach.

Previous studies have reported several predictive markers for risk estimation, such as S100P, ERO1LB, SULF1, ITGA2, GPRC5A, ACTN4, LMO2, p16INK4a, CLPS, COL11A1, GJB2, CTRL, CEL, CPA1, POSTN, PM20D1, and MARCO (Lin et al. 2020; Jiang et al. 2018; Wang et al. 2020; Ho et al. 2020; Watanabe et al. 2015; Nakata et al. 2009; Shi et al. 2021; Gerdes et al. 2002; Lyu et al. 2018; Zhang et al. 2013; Ji et al. 2014). Furthermore, many miRNAs also demonstrate prognostic roles in PCs (Khan et al. 2015; Eid et al. 2021). It is noteworthy that most existing prognostic models for PC involve only one gene or are only related to short-term clinical response (DFS) or long-term clinical effects (OS). Significantly, not all of the biomarkers have been used in the clinic or evaluated for their roles in predictions for immunotherapy. In the present study, we first presented a comprehensive profiling of the tumor, tumor microenvironment, and tumor immune status with 1,833 RNA targets of PC tissue, using the GeoMx CTA Panel. We found five significantly upregulated markers in the PC tissue, including LAMB3, FN1, KRT17, KRT19, and ANXA1. The KEGG pathway enrichment analysis of the cancer tissue included laminin interactions, degradation of the extracellular matrix, extracellular matrix organization, and more, all of which were highly associated with the upregulated genes. The expression pattern of LAMB3, FN1, KRT17, KRT19, and ANXA1 was confirmed by the TCGA dataset. In the 178 PC patients, the LAMB3, FN1, KRT17, KRT19, and ANXA1 were also upregulated in cancer tissue, compared with adjacent tissues and normal tissues. Significantly, survival analysis demonstrated high expression of LAMB3, FN1, KRT19, and ANXA1, but KRT17 was associated with a shorter DFS and OS. The DFS benefits in the low expression of LAMB3, FN1, KRT19, and ANXA1 group indicate that these markers can be used to predict the treatment efficacy of surgery, and the treatment approaches of patients with high expression of LAMB3, FN1, KRT19, and ANXA1 may need to be improved. Further studies are necessary to examine this issue.

Previous studies have demonstrated that LAMB3 could mediate cell cycle arrest and apoptosis in PC cells and alter the proliferative, invasive, and metastatic behaviors of PC by regulating the PI3K/Akt signaling pathway (Zhang et al. 2019; Huang et al. 2020). Inhibition of LAMB3 abrogated the tumorigenic effects of PI3K/Akt signaling pathway activation. Our study found that LAMB3^hi^ PCs upregulated the PI3K/Akt signaling pathway, which is consistent with the findings of the previous study. However, the prognostic role of LAMB3 in distinct stages and treatment groups of PC patients is still unclear. In this study, we found that LAMB3 is significantly upregulated and selectively expressed in PC tissue. High expression of LAMB3 was associated with shorter DFS and OS, suggesting its use as a predicting indicator. Significantly, the univariate analysis and multivariate Cox analysis revealed that LAMB3 can be a negative prognostic indicator for both DFS and OS. This data indicates that LAMB3 can predict the treatment efficacy of surgery and the treatment efficacy of first-line therapy. Immunotherapy has been become the main treatment option for PC; however, it is still unknown whether LAMB3 expression can predict ICB response. Previous studies have confirmed that ICB treatment response can be predicted with TIDE scores (Jiang et al. 2018; Wang et al. 2020), but, with this method, we found that there was no significant difference in TIDE scores of samples with high and low expression of LAMB3. In conclusion, our results show that LAMB3 can be a predictor indicator for the DFS and OS of PC patients.

The adhesive extracellular matrix protein, fibronectin with its integrin receptors play important roles at several stages of multiple tumors development (Erdogan et al. 2017; Glasner et al. 2018; Liang et al. 2020; Liu et al. 2020). In a pivotal study, *Cristina P.R. Xavier* et al*.* reported that FN1 is the most abundant cargo protein released by human macrophage extracellular vesicles (EVs), and it is correlated with lower OS of PC patients (Xavier et al. 2021). Other studies have also demonstrated that FN1 has an unfavorable prognostic impact for PC patients (Akiyama et al. 1995; Munasinghe et al. 2020; Hiroshima et al. 2020). In addition, Hu et al. after performing a immunohistochemical analysis on 138 PC patients, reported that stromal FN1 expression was not associated with long-term survival (Hu et al. 2019). In our study, we used the GeoMx CTA Panel to perform a 1833 targets analysis that included the tumor, tumor microenvironment, and tumor immune status of PC patients. We identified FN1 precisely as the differentially expressed targets in PCs. The increased expression of FN1 in PCs was confirmed by the TCGA dataset, which showed the same expression pattern. Furthermore, high expression of FN1 is highly associated with shorter DFS, OS, and resistance to immunotherapy compared with low expression of FN1 in PC patients. Co-expression networks are significant for the development of cancer (Zhou et al. 2019). Significant transcriptional differences were found in PC patients with high expression of FN1. The top upregulated genes included COL11A1, (Jia et al. 2016) MMP11 (Zhang et al. 2020), MARCO (Shi et al. 2021), GJB2 (Zhou et al. 2019), CD163 (Shi et al. 2021), and CCN4 (Banerjee et al. 2016) (Additional file 3: Table S3) in the high expression of FN1 group, which were highly correlated with a poor prognosis of patients with PC. The enriched KEGG pathway included ECM-receptor interaction and focal adhesion, which was correlated with the regulation of FN1, and FN1 increased cell proliferation and enhanced chemoresistance in PC cells (Miyamoto et al. 2004). Collectively, our results indicate that FN1 can be a biomarker for predicting short-term clinical response, long-term clinical effects, and clinical response to immunotherapy.

Keratins (KRTs) are intermediate filament proteins, responsible for the structural integrity of epithelial cells and markers of epithelial tissue, activating signaling networks that regulate cell migration, invasion, metastasis, cell cycle, and apoptosis (Coulombe and Wong 2004; Hendrix et al. 1996; Saha et al. 2017). KRT19 is a known prognostic biomarker for multiple cancer types, including PCs, and increased KRT19 was closely correlated with a poor prognosis of patients with PC (Saha et al. 2017; Tang et al. 2014; Yao et al. 2016). Experiments with mice have revealed that Krt19^+^/Lgr5^−^cells are radioresistant cancer-initiating stem cells in the colon and intestine (Asfaha et al. 2015), which can partially explain the poorer prognosis of tumors with higher KRT19 expression. Consistent with this finding, our survival analysis, univariate analysis and multivariate Cox proportional hazards model showed that the KRT19^hi^ group of PC patients had a shorter DFS and OS. In contrast, when we examined the sensitivity of PC patients with high and low expression of KRT19 to immunotherapy, we did not find significant differences.

ANXA1 is a calcium-dependent phospholipid-binding protein considered to play an important role in tumorigenesis in multiple types of cancer, including breast cancer, colorectal cancer, and more (Zhang et al. 2010; Graauw et al. 2010; Liang and Li 2021; Wang et al. 2010). In addition, ANXA1 has been shown to have immunomodulatory effects on T-cells, macrophages, and dendritic cells (Dempsey et al. 2021). Significantly, targeting ANXA1 with humanized antibodies inhibits tumorigenic processes and induces an immune response in tumors with an overexpression of ANXA1 (Dempsey et al. 2021). However, there are no conclusions on the prognostic role of ANXA1 in PC, especially in the prediction of sensitivity to immunotherapy. In the present study, we used multiple analytic approaches, including survival analysis, univariate analysis, multivariate Cox proportional hazards model, and analysis of immune response, all of which indicated the predictive roles of ANXA1 in PC patients. Furthermore, compared with the ANXA1^low^ group of PC patients, the ANXA1^hi^ group exhibited significantly increased upregulated genes. In addition, the enrichment of the KEGG pathway in ANXA1^hi^ PC patients included the PI3K–Akt signaling pathway (Zhang et al. 2019), focal adhesion (Sawai et al. 2005), and ECM—receptor interaction (Hosein et al. 2020), all of which are correlated with the progression of cancers. Collectively, we identified a biomarker that predicts DFS, OS, and sensitivity to immunotherapy in PC. In the future, continued screening of ANXA1 in the PC immune microenvironment could help us to develop novel targets more accurately and provide a theoretical basis for the pathological mechanism of PCs.

Notably, the predictive roles of combined LAMB3, FN1, KRT19, and ANXA1 have never been reported. We found that the combination of LAMB3, FN1, KRT19, and ANXA1 can be used as a biomarker to predict DFS in PC. In addition, the combination of LAMB3, KRT19, and ANXA1 can be considered as a biomarker to predict OS in PC.

## Conclusions

However, this study has limitations. We only evaluated 1833 targets in one PC patient. More studies should be conducted to confirm our results and early or late stage pancreatic cancer patients’ samples should also be used to evaluate the differences. In addition, the way that LAMB3, FN1, KRT19, and ANXA1 regulate the prognosis of PCs should be studied further. Nevertheless, the expression of LAMB3, FN1, KRT19, and ANXA1 are still effective predictors of PC prognosis. Furthermore, FN1 and ANXA1 can be predictors of the efficacy of immunotherapy in PC. Last, independent studies are required to confirm the findings of this study.

## Supplementary Information

**Additional file 1: Figure S1.** (A) Technical signal QC reveals raw reads, aligned reads percentage, and sequencing saturation. (B) Technical background QC indicates no template control count (NTC count) and negative probe count. (C) DSP parameters demonstrate the nuclei counts and surface area. (D) Target QC was measured to determine the LOQ. LOQ = GeoMean (NegProbe) × GeoSD (NegProbe)threshold. (E) Probe outlier QC demonstrates the low outlier detection and Grubbs outlier test.

**Additional file 2: Figure S2.** (A) Volcano map indicating the differentially expressed genes in LAMB3hi and LAMB3low groups of PC patients. (B) The upregulated KEGG pathways in the LAMB3hi group of PC patients. (C) The downregulated KEGG pathways in the LAMB3hi group of PC patients. (D) Volcano map indicating the differentially expressed genes in FN1hi and FN1low groups of PC patients. (E) The upregulated KEGG pathways in the FN1hi group of PC patients. (F) Volcano map indicating the differentially expressed genes in KRT19hi and KRT19low groups of PC patients. (G) The upregulated KEGG pathways in the KRT19hi group of PC patients. (H) The downregulated KEGG pathways in the KRT19hi group of PC patients. (I) Volcano map indicating the differentially expressed genes in ANXA1hi and ANXA1low groups of PC patients. (J) The upregulated KEGG pathways in the ANXA1hi group of PC patients. (K) The downregulated KEGG pathways in the ANXA1hi group of PC patients.

**Additional file 3. **The upregulated and downregulated genes in the LAMB3^hi^ group compared with the LAMB3^low^ group.

**Additional file 4. **The expression of the 1833 targets from the paracancer tissue and the cancer tissue by DSP analysis.

**Additional file 5. **The upregulated and downregulated genes in the FN1^hi^ group compared with the FN1^low^ group.

**Additional file 6. **The upregulated and downregulated genes in the KRT19^hi^ group compared with the KRT19^low^ group.

**Additional file 7. **The upregulated and downregulated genes in the ANXA1^hi^ group compared with the ANXA1^low^ group.

## Data Availability

All data generated and materials in the study are included in the present article and supplementary data.

## Abbreviations

AOI: Area of interest
CTA: Cancer transcriptome atlas
DFS: Disease-free survival time
DSP: Digital spatial profiler
EVs: Extracellular vesicles
ICB: Immune checkpoint blockade
KEGG: Kyoto encyclopedia of genes and genomes
OS: Overall survival time
PC: Pancreatic cancer
QC: Quality control
ROIs: Regions of interest
TIDE: Tumor immune dysfunction and exclusion
