# Supplementary figures and images for: Identification and prognostic analysis of biomarkers to predict the progression of pancreatic cancer patients
Source: Mol Med. 2022 Apr 15;28:43. doi: 10.1186/s10020-022-00467-8 (PMC9013045; doi:10.1186/s10020-022-00467-8)

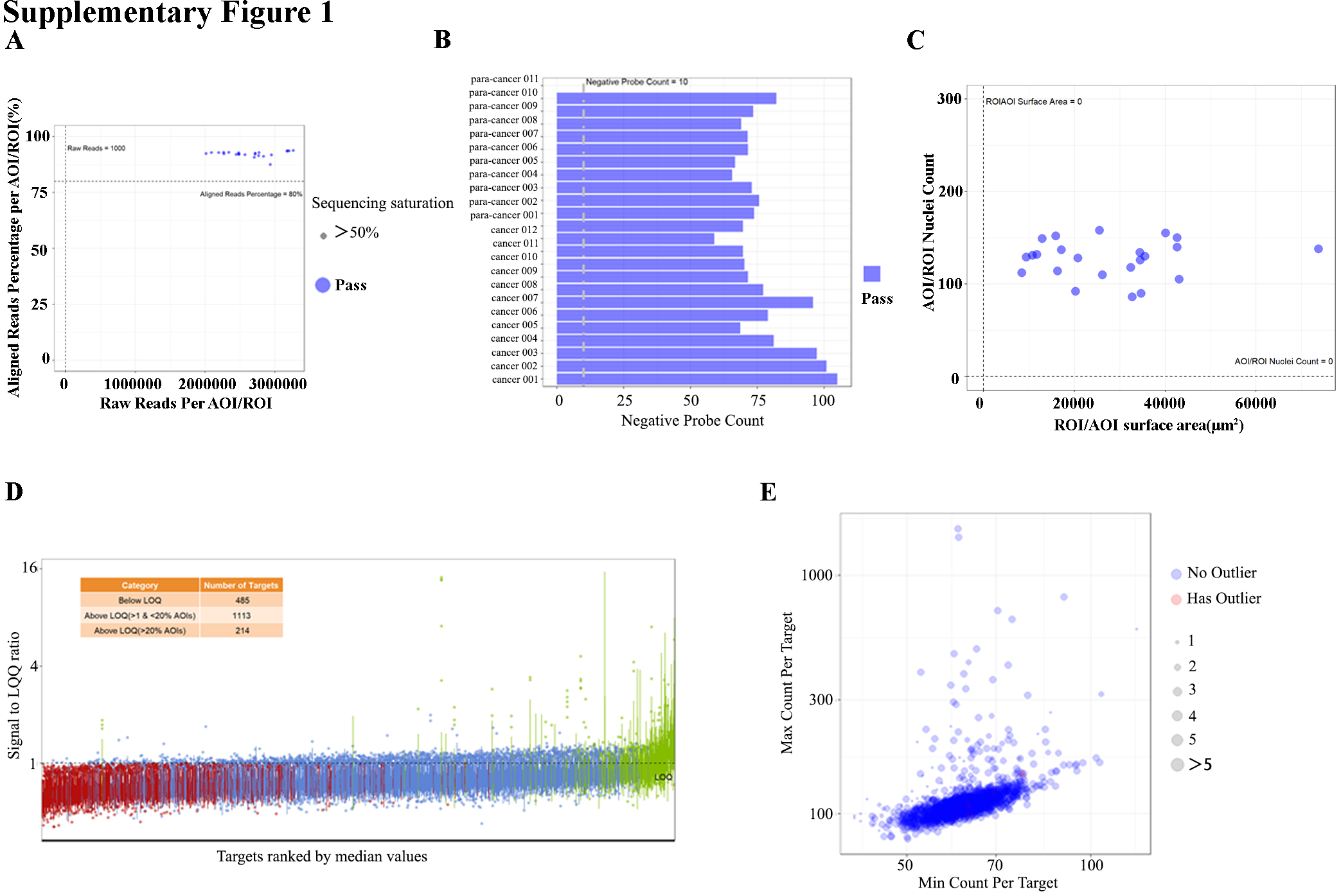

Supplement: Supplementary file 1 — Additional file 1: Figure S1. (A) Technical signal QC reveals raw reads, aligned reads percentage, and sequencing saturation. (B) Technical background QC indicates no template control count (NTC count) and negative probe count. (C) DSP parameters demonstrate the nuclei counts and surface area. (D) Target QC was measured to determine the LOQ. LOQ = GeoMean (NegProbe) × GeoSD (NegProbe)threshold. (E) Probe outlier QC demonstrates the low outlier detection and Grubbs outlier test. [file 10020_2022_467_MOESM1_ESM.tif]

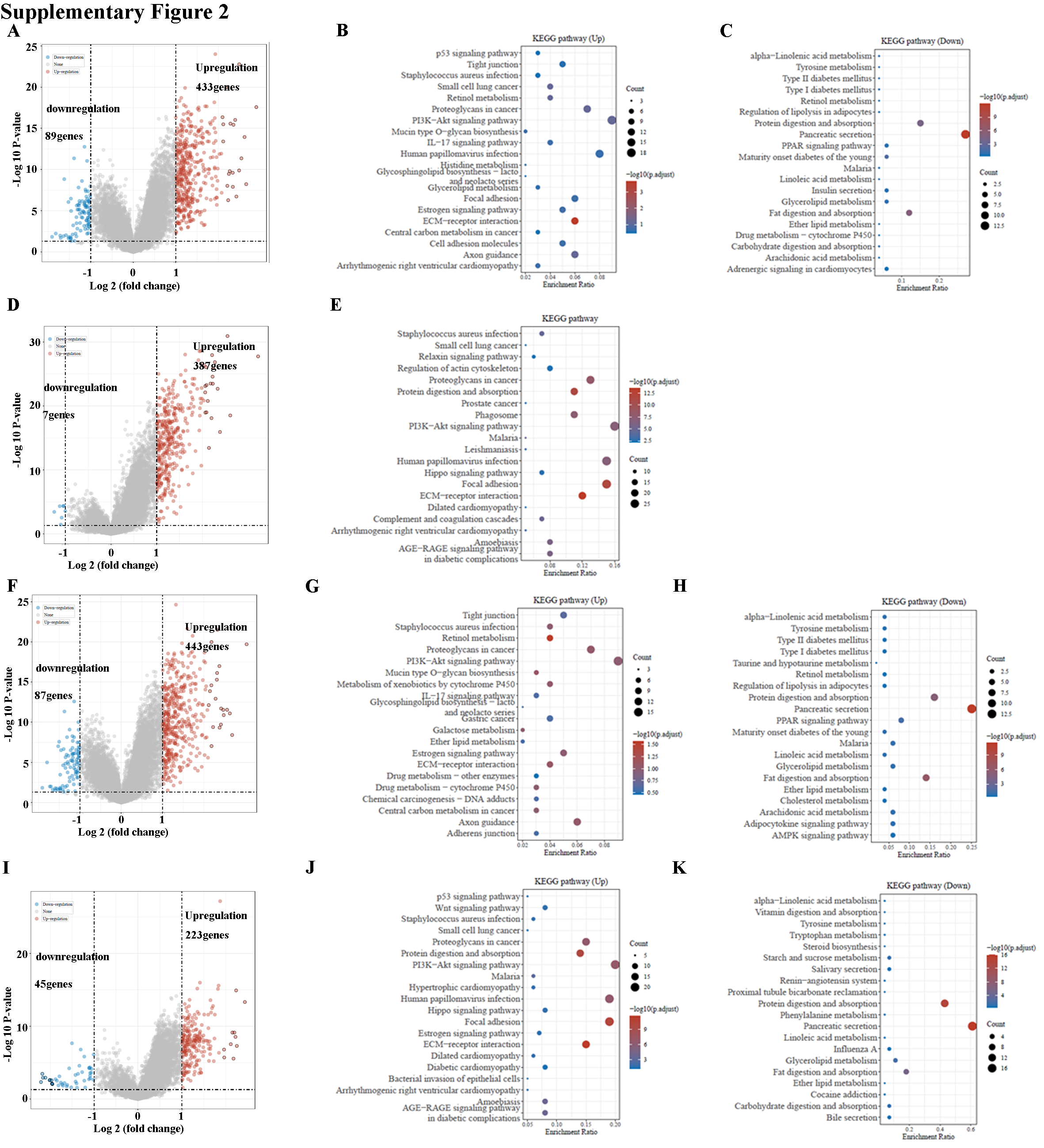

Supplement: Supplementary file 2 — Additional file 2: Figure S2. (A) Volcano map indicating the differentially expressed genes in LAMB3hi and LAMB3low groups of PC patients. (B) The upregulated KEGG pathways in the LAMB3hi group of PC patients. (C) The downregulated KEGG pathways in the LAMB3hi group of PC patients. (D) Volcano map indicating the differentially expressed genes in FN1hi and FN1low groups of PC patients. (E) The upregulated KEGG pathways in the FN1hi group of PC patients. (F) Volcano map indicating the differentially expressed genes in KRT19hi and KRT19low groups of PC patients. (G) The upregulated KEGG pathways in the KRT19hi group of PC patients. (H) The downregulated KEGG pathways in the KRT19hi group of PC patients. (I) Volcano map indicating the differentially expressed genes in ANXA1hi and ANXA1low groups of PC patients. (J) The upregulated KEGG pathways in the ANXA1hi group of PC patients. (K) The downregulated KEGG pathways in the ANXA1hi group of PC patients. [file 10020_2022_467_MOESM2_ESM.tif]
